# Application of Point-of-care Ultrasound for Screening Climbers at High Altitude for Pulmonary B-lines

**DOI:** 10.5811/westjem.2022.11.54300

**Published:** 2023-02-09

**Authors:** Shadi Lahham, John Moeller, Heesun Choi, Chanel Fischetti, Toby Myatt, Nicholas Bove, Soheil Saadat, Proma Mazumder, Isabel M. Algaze Gonzalez, Ami Kurzweil, John C. Fox

**Affiliations:** * Kaiser Permanente Orange County, Department of Emergency Medicine, Anaheim, California; † Dartmouth-Hitchcock Medical Center, Department of Emergency Medicine, Lebanon, New Hampshire; ‡ Kingman Regional Medical Center, Department of Emergency Medicine, Kingman, Arizona; § Midwestern University AZCOM, Department of Emergency Medicine, Glendale, Arizona; ¶ Brigham and Women’s Hospital, Department of Emergency Medicine, Boston, Massachusetts; || Touro University Nevada College of Osteopathic Medicine, Clark County, Nevada; # University of California, Irvine, Department of Emergency Medicine, Orange, California; ** Harvard Medical School, Lecturer in Emergency Medicine, Boston, Massachusetts; †† Eisenhower Health, Department of Emergency Medicine, Rancho Mirage, California

## Abstract

**Introduction:**

High-altitude pulmonary edema (HAPE) occurs as a result of rapid ascent to altitude faster than the acclimatization processes of the body. Symptoms can begin at an elevation of 2,500 meters above sea level. Our objective in this study was to determine the prevalence and trend of developing B-lines at 2,745 meters above sea level among healthy visitors over four consecutive days.

**Methods:**

We performed a prospective case series on healthy volunteers at Mammoth Mountain, CA, USA. Subjects underwent pulmonary ultrasound for B-lines over four consecutive days.

**Results:**

We enrolled 21 male and 21 female participants. There was an increase in the sum of B-lines at both lung bases from day 1 to day 3, with a subsequent decrease from day 3 to day 4(*P*<0.001). By the third day at altitude, B-lines were detectable at base of lungs of all participants. Similarly, B-lines increased at apex of lungs from day 1 to day 3 and decreased on day 4 (*P*=0.004).

**Conclusion:**

By the third day at 2,745 meters altitude, B-lines were detectable in the bases of both lungs of all healthy participants in our study. We assume that increasing the number of B-lines could be considered an early sign of HAPE. Point-of-care ultrasound could be used to detect and monitor B-lines at altitude to facilitate early detection of HAPE, regardless of pre-existing risk factors.

## INTRODUCTION

High-altitude illness (HAI) is a spectrum of pathology including acute mountain sickness, high-altitude cerebral edema, and high-altitude pulmonary edema (HAPE).^1^,^2^ Illness occurs due to acute ascent to altitude faster than the acclimatization processes of the body.^3^–^5^ High-altitude illness generally begins at an elevation of 2,500 meters above sea level.^3^–^5^ The most life-threatening form of HAI is HAPE, which is the result of an abnormal development of fluid within the lungs. High-altitude pulmonary edema typically occurs after rapid accent to altitudes greater than 2,500 meters above sea level.^6^ Symptoms can range from fatigue, nonproductive cough, and dyspnea on exertion to more severe manifestations, which may include frothy sputum production and even respiratory distress.^3^,^7^

The use of point-of-care ultrasound (POCUS) in evaluating interstitial edema has been shown to be highly sensitive and specific for the evaluation of both cardiac and non-cardiac pulmonary edema.^8^–^12^ Previous studies have also shown an association between geographical elevation and the development of non-cardiac pulmonary edema on ultrasound through identification of B-lines (previously “comet tails”).^13^–^15^ Advances in portability and the reduced cost of POCUS units have introduced an opportunity for its application in screening for subclinical interstitial edema at altitude. Our objective in this study was to determine the prevalence and trend of developing B-lines at 2,745 meters above sea level among healthy visitors over four consecutive days.

## METHODS

### Study Setting and Population

We performed a prospective case series using a convenience sample of healthy volunteers between March 4–7, 2019. Subjects were recruited at Mammoth Mountain, CA, where they presented for an educational conference. The site of enrollment was the base of Mammoth Mountain, which is approximately 2,745 meters above sea level. Inclusion criteria were as follows: being older than 18 years; arriving from sea level prior to arrival at the mountain; and planning to present at altitude for at least four days. Exclusion criteria included pregnancy, presence at 1,500 meters altitude or higher at any point within 14 days of study enrollment, a history of pulmonary edema, lung cancer, congestive heart failure, pulmonary hypertension, pulmonary embolism, chronic obstructive pulmonary disease, and/or pneumonia or influenza within 30 days.

All subjects were approached at Mammoth Mountain within 24 hours of arrival and given a study information sheet explaining the purpose of the study. Both verbal and written consent was obtained. The study was approved by the local institutional review board.

### Data Collection

Subjects were given a data collection sheet and asked to report age, gender, medical history, and history of chest trauma. A POCUS was performed on subjects using a Mindray TE7 (Mindray Corp, Shenzhen, China) ultrasound machine with a phased-array 2–5 megahertz transducer in the pulmonary setting. Subjects were scanned in a seated, upright position in the sagittal orientation.

All pulmonary scans consisted of four lung ultrasound images (windows): one mid-clavicular scan at the third intercostal space (apical) of each hemithorax, as well as one posterior-axillary scan at the fourth/fifth intercostal space (base) of each hemithorax (right and left). We counted and recorded the number of B-lines visualized within a single, four-second video clip lung window. Subjects were re-scanned at 24-hour intervals over the course of four consecutive days in the exact same location. Images were obtained by two ultrasound-fellowship trained emergency physicians under supervision of the principal investigator. We summed up the total number of B-lines at the bases of both lungs and observed the change of this discrete variable across days. We also summed up the total B-lines at the apical lung fields of both lungs and reported the change of this discrete variable over the time.

**Population Health Research Capsule**

What do we already know about this issue?

*High-altitude pulmonary edema (HAPE) can occur at 2,500 meters altitude. The treatment is descent, which can be important to plan for in resource-limited settings*.

What was the research question?

*Our goal was to find the trend of developing B-lines at 2,745 meters above sea level among healthy visitors over four consecutive days*.

What was the major finding of the study? Major comparison with P-value and confidence interval

*The median number of B-lines at the bases of both lungs rose from zero in day 1 to three in day 3 and then dropped to two in day 4 (P<0.001)*.

How does this improve population health?

*Point-of-care ultrasound can be used to detect early stages of HAPE in asymptomatic climbers. Early detection affords the opportunity for timely intervention*.

### Statistical Analysis

We performed statistical analysis using SPSS Statistics for Windows, version 23.0 (IBM Corp, Armonk, NY). Age is reported as mean, standard deviation (SD) and median. The sum of B-lines at the bases of both lungs, and also the sum of B-lines at the apexes of lungs are reported as median and interquartile range (IQR). We used Friedman’s test to determine the statistical significance of change in the sum number of B-lines over the four-day time frame, considering the repeated measure structure of data. We used Pearson’s chi square test to compare proportion of subjects with zero B-lines across days. Type I error level was set to 0.05.

## RESULTS

**Figure 1:**
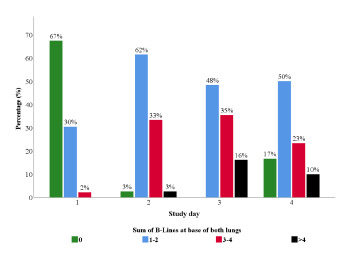
Distribution of the sum of B-lines at the base of both lungs over four days at altitude.

**Figure 2:**
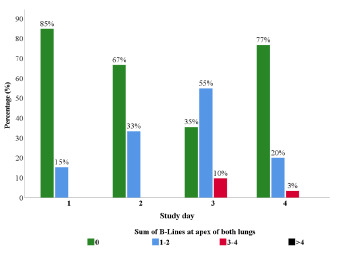
Distribution of the sum of B-lines at the anterior apex of both lungs over four days at altitude.

We enrolled 21 (50%) male participants with the mean age of 35.8 (SD 10.66, median 33) and 21 (50%) female participants with the mean age of 43.4 (SD 13.74, median 40). Figures 1 and 2 illustrate the distribution of the sum of B-lines at the base and apex of both of each subject’s lungs, respectively. The percentage of patients with zero B-lines in the bases of both lungs decreased from 67% to 3% to 0% within the first three days of the study (Figure 1) (*P*< 0.001). In contrast, the percentage of participants with B-lines in all other B-line categories (1–2, 3–4 and >4) increased within the first three days of the study (*P*< 0.001). On day 4, there was a decrease in the percentage of subjects with B-lines >0 (*P*=0.02). The median number of B-lines at the bases of both lungs rose from zero (IQR 0–0) in day 1 to three (IQR 2–4) in day 3 and then dropped to two (IQR 1–3) in day 4. The change in sum of B-lines at the lung bases was statistically significant (*P*<0.001).

The change in the apical lung fields over time was similar to those observed in the lung bases (Figure 2). There was an increase in the sum of B-lines at both lung apices from day 1 to day 3, with a decrease from day 3 to day 4. The percentage of patients with no B-lines in the lung apices decreased from 85% to 67% to 35% within the first three days of the study (*P*< 0.001). The percentage of patients with 1–2 B-lines increased from 15% in day 1 to 55% on day 3 and then decreased to 20% by day 4 (*P*=0.001). Again, the percentage of participants with no B-lines did increase after 72 hours at altitude from 35% to 77% (*P*=0.001). The median number of B-lines at the apical fields of both lungs rose from zero (IQR 0–0) in day 1 to one (IQR 0–2) in day 3 and then dropped to zero (IQR 0–0) in day 4. The change in sum of B-lines at the apical lung fields was statistically significant (*P*=0.004).

## DISCUSSION

The objective of our study was to find out the potential application of POCUS in early detection of HAPE and to identify the prevalence and trend of B-lines among healthy climbers. We detected B-lines at the bases of the lungs of all subjects by the third day at altitude. The participants were healthy volunteers with no pre-existing symptoms or related medical conditions. This indicates that POCUS could be considered for screening HAPE even if there is no significant risk factor. We did not intend to determine what percentage of participants ultimately developed clinical HAPE, but we assume increasing number of B-lines could be considered an early sign of HAPE.

Early detection of HAPE is important especially before dark when transportation becomes difficult. High-altitude pulmonary edema can occur at 2,500 meters altitude. Risk factors for HAPE include rapid ascent, higher altitude, and prior development of HAPE.^6^^,17,19^ Our data illustrates a peak in the number of B-lines at 72 hours, which also coincides with the expected presentation timeline of HAPE. On day 4, the decline in the number of B-lines within both lung locations may have occurred in the setting of acclimatization.

We attempted to control for some sources of variance in the B-lines as follows: By excluding pre-existing pulmonary conditions or infectious symptoms prior to enrollment, we controlled for any factors or pathology outside those caused by changes in altitude. We also considered the possibility of trauma as a cause for B-lines. Pulmonary contusions, as the result of direct/indirect mechanical trauma, have been shown to cause isolated B-lines on pulmonary ultrasound^16^; thus, monitoring B-lines in climbers with chest trauma may be another application of POCUS at altitude.

We did not evaluate the relationship between development of B-lines at altitude and clinical HAPE. Prior studies have defined clinical, non-cardiogenic pulmonary edema in the setting of HAPE by a B-line score.^10^ Yang et al reported sensitivity and specificity of pulmonary ultrasound for HAPE as 98.4% and 90.9%, respectively.^10^ Future large-scale studies are needed to determine whether POCUS can be used as a method of predicting which individuals will potentially develop life-threatening pulmonary edema at altitude.

## LIMITATIONS

Our sampling strategy and exclusion criteria limit generalizability to a young and healthy population. Many participants spent recreational time performing physically demanding activities such as skiing or snowboarding. The intensity of activity required for skiing or snowboarding generally predicts a certain level of cardiopulmonary fitness. Future large-scale studies are needed to determine whether the observed trends in B-lines exist or become more exaggerated under higher altitude conditions. Additionally, many factors were self-reported in data collection and could not be validated.

## CONCLUSION

By the third day at 2,745 meters altitude, B-lines were detectable in the bases of lungs of all healthy participants in our study. Point-of-care ultrasound could be used to detect and monitor B-lines at altitude to facilitate early detection of high-altitude pulmonary edema, regardless of pre-existing risk factors. The importance of these findings and the relationship with further development of altitude illness is yet to be studied.
